# Retraction: CircNF1-419 improves the gut microbiome structure and function in AD-like mice

**DOI:** 10.18632/aging.204905

**Published:** 2023-07-30

**Authors:** Chen Diling, Qi Longkai, Guo Yinrui, Liu Yadi, Tang Xiaocui, Zhu Xiangxiang, Zeng Miao, Li Ran, Shuai Ou, Wang Dongdong, Xie Yizhen, Yuan Xujiang, Burton B. Yang, Wu Qingping

**Affiliations:** 1 State Key Laboratory of Applied Microbiology Southern China, Guangdong Provincial Key Laboratory of Microbial Culture Collection and Application, Guangdong Open Laboratory of Applied Microbiology, Guangdong Institute of Microbiology, Guangdong Academy of Sciences, Guangzhou 510070, China; 2 Guangdong Pharmaceutical University, Guangzhou 510006, China; 3 Guangxi University of Chinese Medicine, Nanning 530023, China; 4 Academy of Life Sciences, Jinan University, Guangdong Province, Guangzhou 510000, China; 5 Chengdu University of Traditional Chinese Medicine, Chengdu 610075, China; 6 Department of Physiology, Shantou University Medical College, Shantou 515063, China

**Keywords:** circular RNAs, microbiome-gut-brain axis, dietary interventions, gut microbiome, therapeutic markers

**This article has been retracted:** Aging has completed its investigation of this paper. We confirmed that the fecal transplantation data from the model group (the APP/PS1 mice) presented in **Figure 2** (panels **2C-2F**) were reused in a Brain Research article [1] published two years after this one. In addition, we found Western blot duplication presented in **Figure 3**, panels **3C** (Expression of the proteins AChE, AMP, CHRNA1 and CHRNB1 in the brain tissues of SAMP8 mice after infection of over-expressing circNF1-419 AAV) and **3E** (Expression of the proteins AChE, AMP, CHRNA1 and CHRNB1 in the brain tissues of 2-month-old mice after infection of over-expressing circNF1-419 AAV). All fourteen authors were notified and all agree to the retraction, though only twelve signed the retraction letter. Two authors, Burton B. Yang and Wu Qingping, stated that they did not have an opportunity to review the work or the manuscript before its submission. Dr. Yang also stated that he did not make a contribution to the paper, and his affiliation listed in the article does not correspond to his actual affiliation. The unresolved concerns call into question the validity of the reported results and the adherence of this article to the Aging Authorship policy. Therefore, the Aging Editors retract this article.
